# Viral Diversity of Tick Species Parasitizing Cattle and Dogs in Trinidad and Tobago

**DOI:** 10.1038/s41598-019-46914-1

**Published:** 2019-07-18

**Authors:** Stephen Sameroff, Rafal Tokarz, Roxanne Albertha Charles, Komal Jain, Alexandra Oleynik, Xiaoyu Che, Karla Georges, Christine V. Carrington, W. Ian Lipkin, Chris Oura

**Affiliations:** 1 Center for Infection and Immunity, Mailman School of Public Health, Columbia University, New York, USA; 2 School of Veterinary Medicine, The University of the West Indies, St. Augustine, Trinidad and Tobago; 3 Department of Preclinical Sciences, The University of the West Indies, St. Augustine, Trinidad and Tobago

**Keywords:** Viral vectors, Phylogenetics

## Abstract

Ticks are vectors of a wide variety of pathogens that are implicated in mild to severe disease in humans and other animals. Nonetheless, the full range of tick-borne pathogens is unknown. Viruses, in particular, have been neglected in discovery efforts targeting tick-borne agents. High throughput sequencing was used to characterize the virome of 638 ticks, including *Rhipicephalus microplus* (n = 320), *Rhipicephalus sanguineus* (n = 300), and *Amblyomma ovale* (n = 18) collected throughout Trinidad and Tobago in 2017 and 2018. Sequences representing nine viruses were identified, including five novel species within *Tymovirales*, *Bunyavirales*, *Chuviridae*, *Rhabdoviridae*, and *Flaviviridae*. Thereafter the frequency of detection of viral sequences in individual tick species was investigated.

## Introduction

Many factors, including relative promiscuity in host selection and duration of attachment, contribute to the efficiency of ticks as vectors of microbial pathogens^1^. Ticks transmit a wide range of viral, bacterial, and protozoan pathogens to both humans and other animals^2^. Although the public health emphasis for tick-borne diseases has largely focused on bacterial pathogens, there is abundant evidence that viral pathogens are also important.

Ticks are the vectors of several viruses important to human and livestock disease including Powassan virus^3^, tick-borne encephalitis virus^4^, Crimean-Congo hemorrhagic fever virus^5^, Alkhurma hemorrhagic fever virus^6^, Colorado tick fever virus^7^, Kyasanur Forest virus^8^, Louping ill virus^9^, Omsk hemorrhagic fever virus^10^, African swine fever virus^11^, and Nairobi sheep disease virus^12^. With improvements in molecular techniques, novel pathogenic viruses are continually being identified. A recent example is the identification of the novel bunyavirus, severe fever with thrombocytopenia syndrome virus (SFTSV), in 2009^13^. Since its discovery, SFTSV has been associated with 7,419 cases including 355 deaths^14^. Other recently identified tick-borne viruses include Bourbon virus^15^, Heartland virus^16^ and Guertu virus^17^. Recent metagenomic studies of ticks uncovered a wide range of highly divergent viruses that do not meet current traditional classification guidelines, including the identification of a new viral order, *Jingchuvirales*^18–25^.

A total of 23 tick species have been identified in the Caribbean twin-island Republic of Trinidad and Tobago, parasitizing a wide range of reptiles, amphibians, birds, and mammals^26^. *Rhipicephalus sanguineus* and *Rhipicephalus microplus* are particularly important due to their global distribution and association with tick-borne diseases^2,27,28^. *R. sanguineus*, commonly referred to as the brown dog tick, is a three-host tick that primarily feeds on canines but can also feed on cats, rodents, birds, and humans. It has been implicated in the transmission of pathogenic species of *Ehrlichia*, *Babesia*, and *Rickettsia*^27^. *R. microplus*, commonly referred to as the southern cattle tick, is a one-host tick that feeds primarily on cattle, deer, and buffalo and has been linked with the transmission of *Borrelia*, *Anaplasma*, and *Babesia* species^28^. To date, neither species has been implicated in the transmission of a viral pathogen.

Historically, surveillance on ticks and tick-borne diseases has been limited in the Caribbean compared to other regions of the world. Currently employed diagnostic assays consist of polymerase chain reaction (PCR) or serological screening for known bacterial and parasitic agents of livestock or canine diseases^29–32^. Because of limited microbial discovery research in the country, it is unclear whether ticks transmit any viral agents. In Trinidad and Tobago, there is increasing evidence of tick-borne agents. Cases of a southern tick associated rash illness (STARI)-like illness, a suspected tick-borne rash, have occurred in patients following a tick bite within the country^33^. Tacaribe virus, originally isolated from bat and mosquito samples from these islands, may actually be a tick-borne agent^34,35^. A lack of surveillance in this region, combined with this new evidence supports the need for an exploratory survey of tick-borne pathogens in Trinidad and Tobago.

The advent of high throughput sequencing platforms has facilitated research exploring the diverse components of the tick microbiome. An increasing incidence of tick-borne diseases across the world emphasizes the need to further characterize the tick microbiome to uncover novel agents that have the potential to be pathogenic or influence the transmission of known pathogens. Despite a large biodiversity in the Caribbean, research on tick-borne disease has been historically neglected. This study represents the first virome analysis in this region while also surveying the frequency of these viruses within the tick population.

## Results

**Figure 1:**
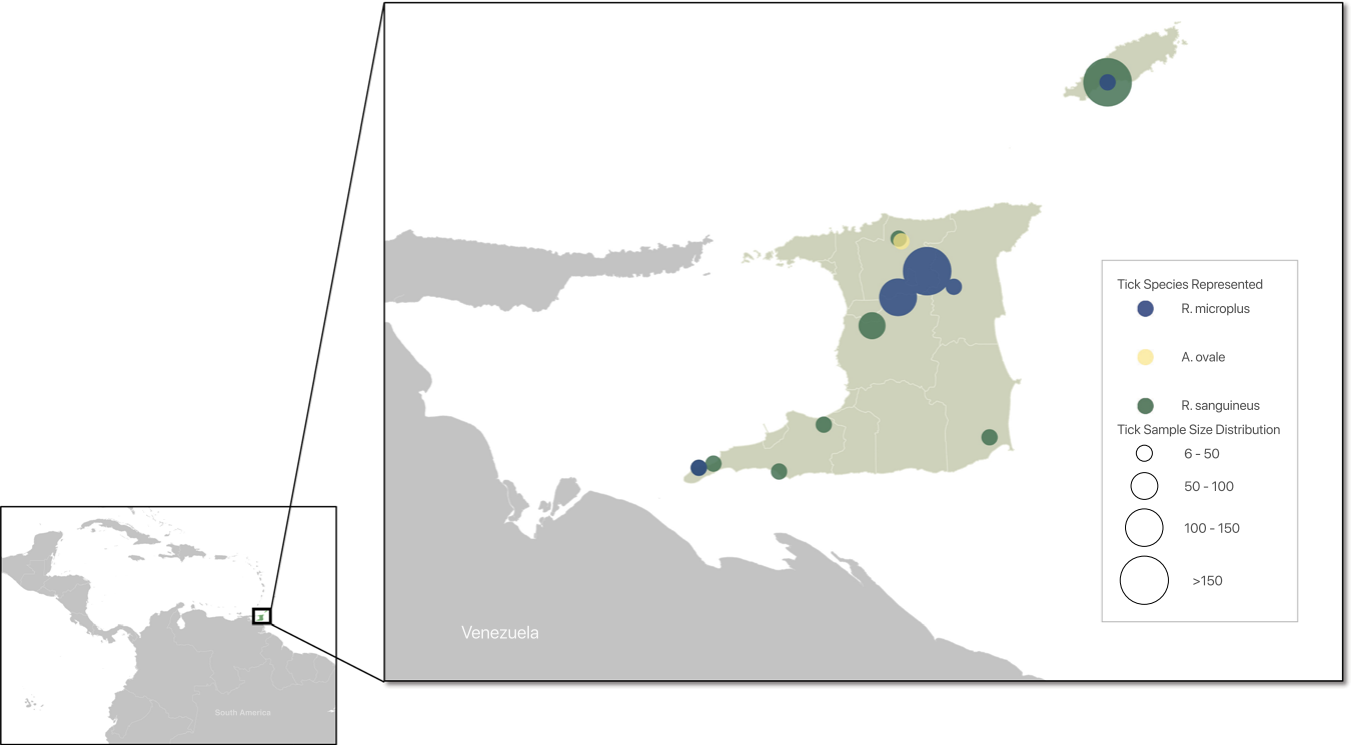
Tick collection numbers by site and species in Trinidad and Tobago. This map was generated using QGIS 3.4.2 using DIVA GIS shape files.

A total of 763 ticks were collected from 15 different sites throughout Trinidad and Tobago (Fig. 1). The collection included ticks from the environment and from 82 different animals. PCR barcoding revealed that 362 ticks were *R. microplus* (removed from 16 cattle), 395 were *R. sanguineus* (55 from the environment and the remainder from 52 dogs), and 18 were *A. ovale* (from 4 hunting dogs). For HTS, 32 pools (16*R. microplus, 15 R. sanguineus, 1 A. ovale)* consisting of 20 ticks each (except the *A. ovale* pool, n = 18) were generated from 638 ticks.

Two lanes of Illumina HiSeq were used to sequence all tick pools (16 tick pools and one negative control per lane) resulting in 682,261,483 raw reads with an average of 20,250,595 (±4,692,632) raw reads per pool. Of these, 37,650,809 (6%) remained for assembly after filtration and host subtraction, which were assembled into 5,042,271 contigs. Of those contigs, 20,924 (0.42%) could be identified as viral sequences through BLASTn or BLASTx. Detailed information on the sequencing results are provided (Supplemental Tables 1 and 2). Nine viral species were identified (five novel) from five viral families (Table 1).

**Table 1:** Viruses identified in Trinidad and Tobago through high throughput sequencing.

Virus	Family	Tick Species	Closest Relative	% Identity (aa)	Prevalence	Genome Length (nt)*
Trinbago virus	Flaviviridae	R. sanguineus R. microplus A. ovale	Bole tick virus 4	86%	24% 3% 5%	16,274
Jingmen tick virus (C)	Flaviviridae	R. microplus	Jingmen tick virus (Kosovo)	95%	46%	(Segment 1) 3,156 (Segment 2) 2,848 (Segment 3) 2,824 (Segment 4) 2,794
Jingmen tick virus (AS)	Flaviviridae	R. microplus	Jingmen tick virus (Kosovo)	94%	6%	(Segment 1) 3,012 (Segment 2) 2,814 (Segment 3) 2,667 (Segment 4) 2,701
Blanchseco virus	Rhabdoviridae	A. ovale	Bole tick virus 2	57%	5%	11,512
Brown dog tick phlebovirus 1	Phenuiviridae	R. sanguineus R. microplus	Bole tick virus 1	59%	78% <1%	(L) 6,614 (S) 1,421
Brown dog tick phlebovirus 2	Phenuiviridae	R. sanguineus R. microplus	Tick phlebovirus	93%	91% <1%	(L) 6,532 (S) 2,093
Lihan tick virus (Trinidad)	Phenuiviridae	R. microplus	Lihan tick virus	99%	90%	(L) 6,495 (S) 1,546
Cattle tick tymovirus-like virus 1	Unclassified	R. microplus	Guarapuaya tymovirus-like 2 (incomplete genome)	89%	3%	6,464
Brown dog tick mivirus 1	Chuviridae	R. sanguineus	Changping mivirus	63%	12%	11,272
Wuhan mivirus (Trinidad)	Chuviridae	R. microplus R. sanguineus	Wuhan mivirus	99%	88% <1%	11,187

*Approximate genome size. All ORFs are complete but the ends were not confirmed as termini.

### Bunyavirales

**Figure 2:**
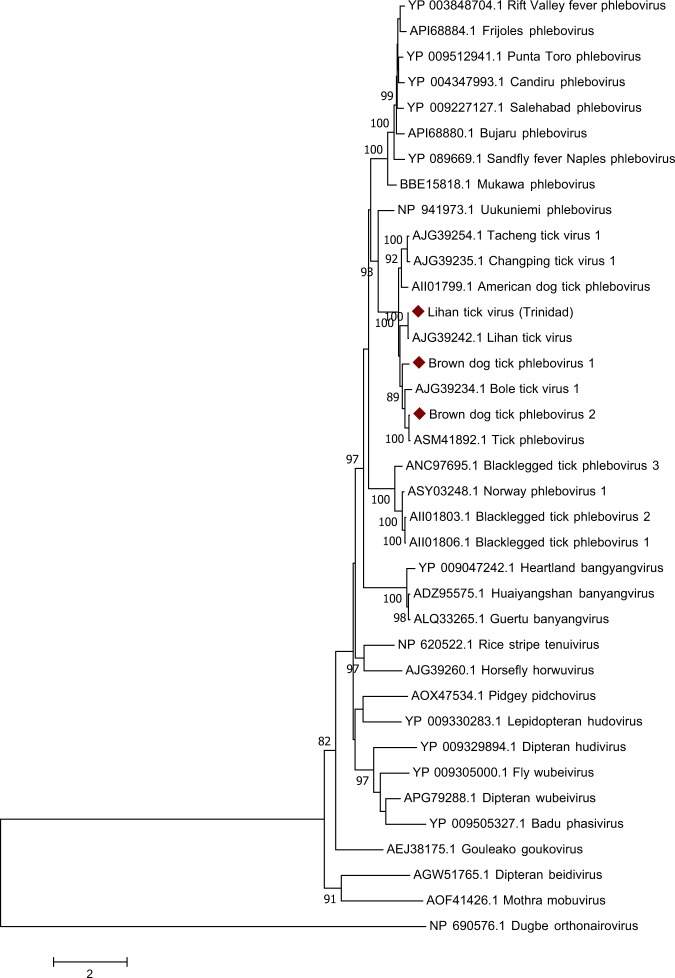
Phylogenetic relationships of *Phenuiviridae* based on a 483-aa fragment of the RdRp that includes the premotif through motif E of the conserved palm domain.

Sequences from three phlebovirus-like viruses, tentatively designated as brown dog tick phlebovirus 1 (BDTPV1), brown dog tick phlebovirus 2 (BDTPV2) and Lihan tick virus-Trinidad (LTV-T), were identified. These viruses have shared similarity in both the L and S segments to known phleboviruses^36^, but lack an M segment, a critical component of the phlebovirus genome that encodes the viral glycoprotein allowing cell entry^37^. Similar viral sequences have been identified in previous tick virome studies^18,20–22,24,25,38^. BDTPV1 is a novel virus with closest similarity (59% amino acid (aa) in the L segment) to Bole tick virus 1, which was recently identified in *Hyalomma asiaticum* ticks in China^19^. BDTPV1 had a prevalence rate of 78% of *R. sanguineus* ticks and was also found in two *R. microplus* ticks (<1%). BDTPV2 had highest similarity (93% aa in the polymerase) to tick phlebovirus identified in *R. bursa* ticks in Turkey^39^. Viral nucleic acid was also found in 91% of *R. sanguineus* ticks and was also detected in two *R. microplus* ticks (<1%). LTV-T was highly similar (99% aa similarity to the polymerase) to Lihan tick virus, identified in *R. microplus* ticks in China^19^. LTV-T was highly prevalent in *R. microplus* ticks sampled with a rate of 90%, but was not found in any other tick species. All three viruses identified cluster with many of the other bunyaviruses lacking M-segments and share a common ancestor with the Uukuniemi phlebovirus group (Fig. 2).

### Tymovirales

**Figure 3:**
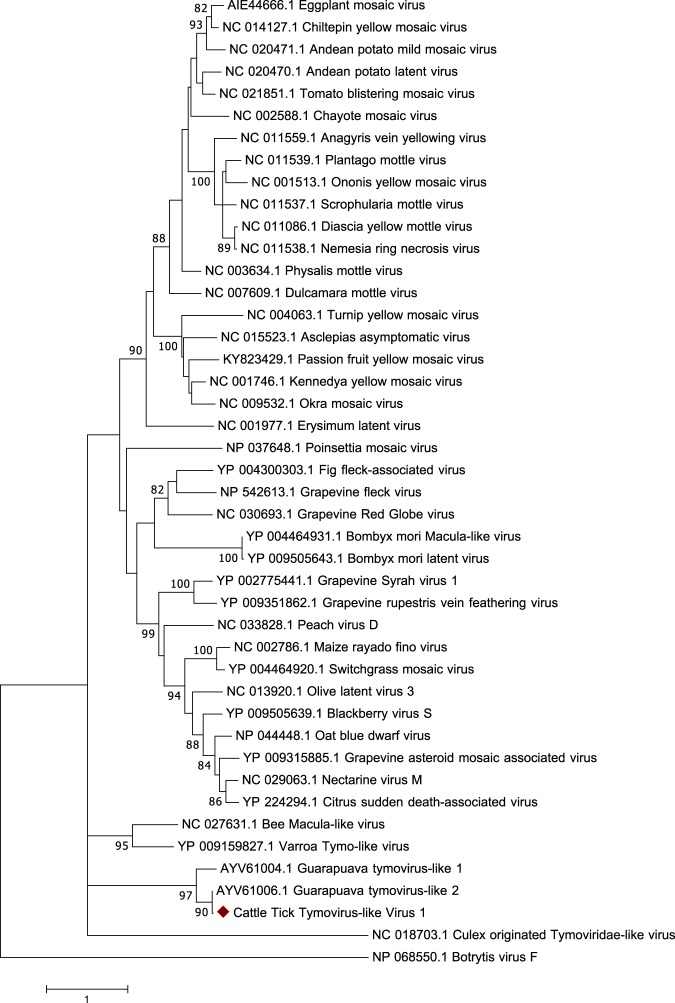
Phylogenetic relationship of *Tymovirales* based on a 1068-aa alignment of the replicase polyprotein.

A single virus with similarity to the order *Tymovirales* was identified and tentatively named cattle tick tymovirus-like virus 1 (CTTV1). This virus was most similar to Guarapuava tymovirus-like 2 (97% aa similarity to the coat protein and 90% aa similarity to the partial polyprotein), which was recently identified in *R. microplus* ticks parasitizing cattle in Brazil^22^. CTTV1 follows the traditional genome organization for viruses within *Tymovirales* with a large polyprotein followed by a small coat protein, however was missing the movement protein that overlaps with the polyprotein. The phylogenetic relationship of CTTV1 and other viruses within the order *Tymovirales* shows that CTTV1 along with Guarapuava tymovirus-like 1 and 2 form a distinct clade separate from other recognized tymoviruses (Fig. 3). CTTV1 was present in only 3% of all *R. microplus* ticks screened, all of which were removed from the same animal, and was not present in either *R. sanguineus* or *A. ovale* ticks.

### Chuviridae

**Figure 4:**
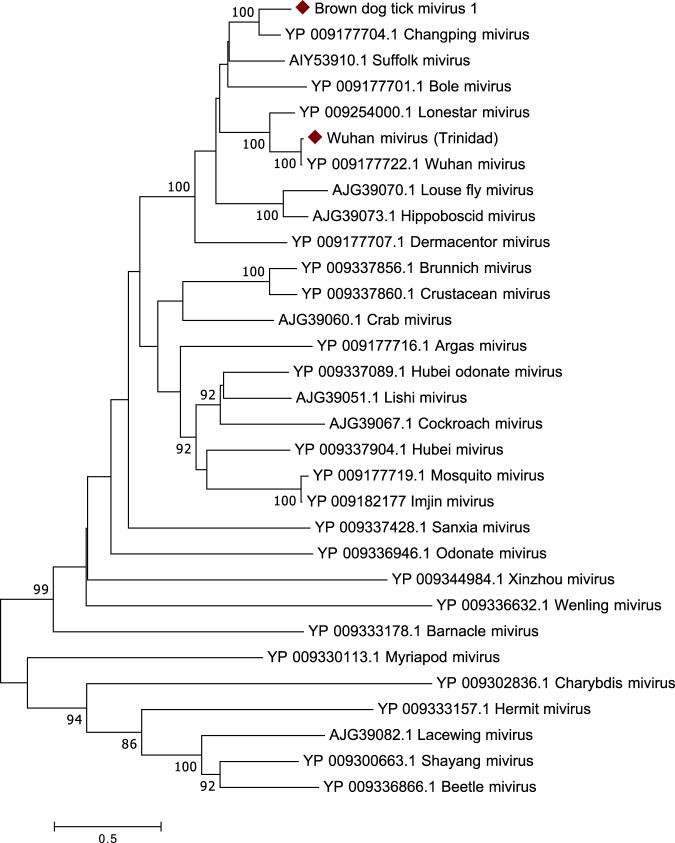
Phylogenetic relationships of *Chuviridae* based on a 377-aa alignment of the RdRp.

Two species of miviruses were identified in this study. The first, identified in *R. microplus*, has high similarity (99% aa) to Wuhan mivirus (WMV)^19^ and was detected in 88% of *the R. microplus* ticks sampled and one *R. sanguineus* sample. The second mivirus, tentatively named brown dog tick mivirus 1 (BDTMV1), was identified in *R. sanguineus* and had 63% aa similarity to the polymerase of Changping mivirus^19^. BDTMV1 was identified in 12% of the *R. sanguineus* individuals and was absent in all other tick species surveyed in this study. Both of WMV (Trinidad) and BDTMV1 had a circular genome organization with 4 open reading frames (ORFs), similar to other tick-borne miviruses and clustered with other known tick-borne miviruses (Fig. 4).

### Rhabdoviridae

**Figure 5:**
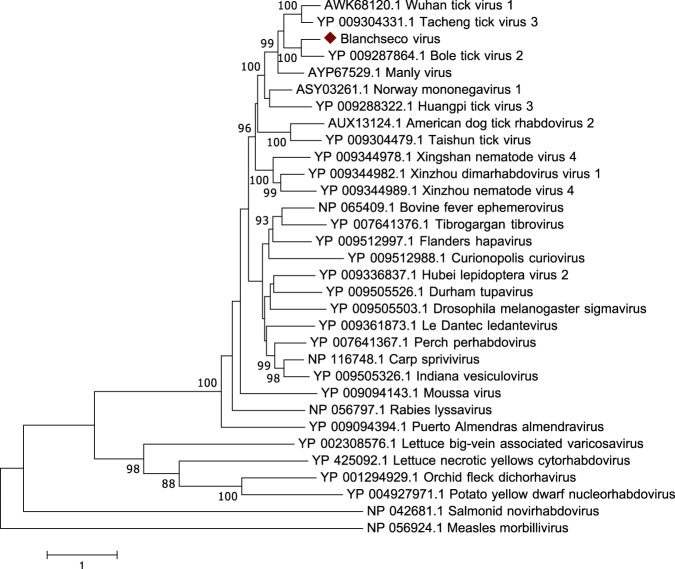
Phylogenetic relationship of *Rhabdoviridae* based on a 1,255-aa alignment of the RdRp.

We identified sequences from a novel rhabdovirus in the one pool of *A. ovale* ticks, tentatively named Blanchseco virus (BCOV). This virus was highly divergent from the most closely related rhabdovirus, Bole tick virus 2 (BTV2)^19^, with only 57% aa similarity in the polymerase. The genome of BCOV has the classical rhabdovirus genome organization of N-P-M-G-L, with no additional large ORFs. Comparison of the RNA-dependent RNA polymerase (RdRp) protein sequence revealed that Blanchseco forms part of a monophyletic cluster with other tick-borne rhabdoviruses within the dimarhabdovirus super group (Fig. 5). BCOV was found in only one out of 18 *A. ovale* ticks (6%).

### Flaviviridae

**Figure 6:**
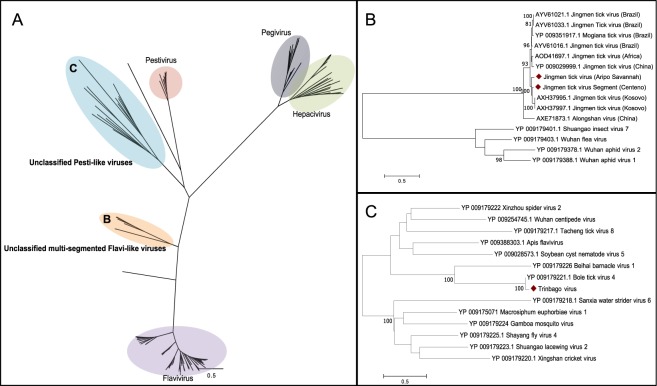
Phylogenetic relationships of *Flaviviridae* based on an alignment of a 655-aa conserved region of the NS5. (**A**) Phylogenetic tree of all species belonging to *Flaviviridae*. (**B**) Close up view of the unclassified multi-segmented Flavi-like group. (**C**) Close up view of the unclassified Pesti-like group.

Sequences from two viruses with similarity to viruses within the *Flaviviridae* family were identified. The first, tentatively named Trinbago virus (TBOV), is a novel virus with an overall 86% aa similarity to the polyprotein of Bole tick virus 4 (BTV4), a virus recently identified in China^38^. TBOV shares greatest amino acid similarity to viruses within the genus *Pestivirus* (<30% within the nonstructural protein 3 (NS3) and nonstructural protein 5 (NS5) peptides). Phylogenetic analysis of the NS5 protein indicates that TBOV clusters with a group of viruses that form a distinct clade outside *Pestivirus*, suggesting that they represent a novel genus within *Flaviviridae* (Fig. 6). TBOV was the only virus from this study identified in all three tick species, with a prevalence of 24% in *R. sanguineus*, 3% in *R. microplus*, and 6% in *A. ovale*.

The second flavivirus clusters with the Jingmen tick virus (JTV) group, a recently identified group of multi-segmented *Flaviviridae*-like viruses^40^. Complete coding regions of two separate genotypes were assembled and tentatively named Jingmen tick virus (Centeno) [JTV(C)] and Jingmen tick virus (Aripo Savannah) [JTV(AS)]. Both viruses contained four segments. Segment 1 encodes a NS-5 like protein, segment 2 encodes a putative glycoprotein, segment 3 encodes a NS-3 like protein, and segment 4 encodes the putative VP-2 VP-3 proteins^22,40,41^. JTV(C) and JTV(AS) are 95% aa and 94% aa identical to the next closest JTV within the NS5-like protein, respectively. The multi-segmented *Flaviviridae*-like viruses form two distinct clades with all the viruses within the JTV group forming one monophyletic cluster (Fig. 6). While JTV(C) was identified from ticks collected at multiple geographical regions (both Centeno and Cedros), JTV(AS) was identified only from ticks collected from sites within the Aripo Savannah. The prevalence of JTV (C) was 46% in *R. microplus* ticks, and the prevalence of JTV(AS) was 6%. We did not identify any co-infections with both JTVs.

### Viral richness

Differences in the viral richness between the three tick species were significant at p = 5.70 × 10^−17^, and all three *post hoc* pairwise comparisons were also significant after Bonferroni correction (Table 2).

**Table 2:** Viral richness by tick species.

*Species*	Average number of viruses (individual tick)	Standard deviation
*A. Ovale* ^•♦^	0.11	±0.32
*R. sanguineus* ^•+^	2.18	±0.82
*R. microplus* ^♦+^	2.35	±0.68

^•^Pairwise comparison of *A. ovale* and *R. sanguineus*: p = 1.82 × 10^−14^.

^♦^Pairwise comparison of *A. ovale* and *R. microplus*: p = 1.55 × 10^−14^.

^+^Pairwise comparison of *R. sanguineus* and *R. microplus*: p = 8.24 × 10^−5^.

## Discussion

The focus of this study was on tick species that parasitize companion animals as well as livestock. The close proximity of these animals to humans may increase the risk of zoonotic transmission. Since *R. microplus* is a single-host tick, and the preference of *R. sanguineus* is to hide on building structures and in crevices, using a drag-flag collection method for these species was not practical. Therefore, the sampling strategy chosen was to collect ticks directly from infested animals (cattle and dogs) and their habitat from different regions of the country. This affected collection success, which was higher for *R. sanguineus* and *R. microplus* than for *A. ovale*. Since dogs are not the primary hosts for *A. ovale*, we speculate that these ticks attached to the dogs as they tracked animals through the bush during hunting.

This study characterized the virome of the tick species parasitizing companion animals and livestock in Trinidad and Tobago. Viruses related to those identified in the current study have been identified in other metagenomic tick virome analyses in North America, South America, Asia, and Northern Europe. Similar viruses have been reported to be present in *Ixodes, Dermacentor, Rhipicephalus* and *Hyalomma* ticks, despite the extensive evolutionary distance between these genera suggesting a possible symbiotic relationship for some of these viruses with their tick hosts. Analyses of additional tick species from diverse geographical areas will help in confirming the evolutionary association of these viruses with their tick hosts.

In previous work, phlebovirus-like viruses that were distinct from other bunyaviruses in that they did not have a glycoprotein-encoding M-segment were also discovered^24,25^. Other groups have subsequently identified similar viruses in ticks from Europe, South America, North America, and Asia^19–21^. We found three such viruses in this study. Lacking the glycoprotein for cellular attachment, these phlebovirus-like viruses currently defy traditional classification, and further work is required to determine if they can form virions, and complete transmission cycles without a glycoprotein^37^. Because of their high prevalence, high similarity across broad geographic regions, and ability to be transovarially transmitted^24^, these viruses may represent viral endosymbionts that are not under selective immunological pressure. One possible scenario is that they are helper-dependent viruses, requiring assistance from another microbe or even the host in order to gain cellular entry. Curiously, previous viral metagenomic studies of *Amblyomma americanum*, did not detect any M segment-less phlebovirus-like viruses^24,25^. They were also absent in the 18 *A. ovale* ticks analyzed in this study. These results suggest that *Amblyomma* ticks may have lost these viruses over time, and further investigations into ticks from other geographical locations would be required to test this hypothesis.

Miviruses (family *Chuviridae*) were first discovered in ticks and novel species have been frequently identified in tick metagenomic studies^18–22,24,25^. Miviruses have only recently been classified by the International Committee on Taxonomy of Viruses (ICTV). They belong to the order *Jingchuvirales*, and were originally associated with viruses within the order *Mononegavirales. Jingchuvirales* display variable genomic organization, including monopartite linear, monopartite circular, and multipartite circular genomes^19^. To date, miviruses have been predominantly identified in arthropods, and in a single nematode. Similar to the phlebovirus-like viruses, they also have high similarity across broad geographic ranges. For example, miviruses identified in both China and Trinidad share 99% similarity when comparing amino acid sequences. At least one species has been reported to be transovarially transmitted and may be endosymbionts^24^.

This study identified a putative highly divergent tymovirus. These viruses are typically associated with plants, where they can cause mosaic disease^42^. Arthropods are presumed to be mechanical vectors, and as a result, tymoviruses are occasionally identified in arthropod metagenomic studies. We speculate that CTTV1, the virus identified in our study, may have been present on the tick cuticle or acquired through the spiracles.

The novel rhabdovirus BCOV was identified in one of 18 ticks removed from canines at a single kennel. *Rhabdoviridae* comprise a wide range of arthropod-borne and zoonotic vertebrate pathogens. Viruses within two genera in this family, *Lyssavirus* and *Ephemerovirus*, can infect the cells of the central nervous system, and cause gradual paralysis^43,44^. At the kennel where all 18 *A*. *ovale* ticks were obtained, one dog suffered from hind limb paralysis and general weakness. It is possible that this dog was suffering from tick paralysis, a reaction caused by a toxin in the tick’s saliva that can also cause progressive motor paralysis^45^. Alternatively, the paralysis may have been due to central nervous system infection with this novel rhabdovirus.

TBOV clusters with an increasing number of novel viruses within the family *Flaviviridae* that are genetically distinct from flaviviruses in ICTV-recognized genera^38^. These viruses contain NS3 and NS5 domains most similar to viruses within the genus *Pestivirus*. In previous tick metagenomic studies, viruses had a high degree of association to a single host, being rarely detected in multiple tick species^19,21,22,24,25^. The majority of tick-borne viruses are vertically and not horizontally transmitted^46,47^. TBOV was unique in that it was detected in all three tick species we examined. We propose that the ability of TBOV to infect multiple tick species suggests that it is unlikely to be a tick endosymbiont and instead is likely to be acquired from a vertebrate host. Because flaviviruses can cause human and animal disease, the association of TBOV virus with tick-borne illness warrants further investigation.

The Jingmen tick viruses are the only viruses identified in this study that were previously shown to infect a vertebrate host. JTV was first isolated from *R. microplus* ticks and from JTV antibody-positive cattle serum from China^40^ and later in both ticks and blood from cattle in Brazil^22^. Recently, this virus was detected as a co-infection in serum from three patients suffering from Crimean Congo hemorrhagic fever in Kosovo^48^. It was also detected in a red colobus monkey in Uganda^49^. The pathogenicity of JTV is unknown. We detected two strains of JTV in Trinidad and Tobago but they did not occupy the same geographical space. This may be due to cross-immunity within cattle populations, niche separation, or ecological boundaries.

In addition to pursuing virome characterization, we were also interested in examining the relative richness of viral diversity by tick species. Our data show that *A. ovale* ticks harbor fewer viruses than both *Rhipicephalus* species. Each pool of *R. sanguineus* and *R. microplus* included a greater number of viruses than the single *A. ovale* pool. We acknowledge that the limited number of *A. ovale c*ollected may contribute to this result but note precedent for differences in viral diversity by tick species. In a study carried out in the United States, *A. americanum* was found to contained fewer viruses than *Ixodes scapularis* and *Dermacentor variablis* ticks^24^.

The clinical implications of our findings are unclear but provide the foundation required to establish the molecular and serological tools required to investigate the role of tick-borne viruses in diseases of dogs and other animals.

## Methods

### Sample collection

Ticks were collected in Trinidad and Tobago in 2017 and 2018 using convenience sampling, collecting ticks off of the animals they parasitize. Select locations were targeted for collection such as the humane society and animal welfare organizations. Sampling was also conducted in locations at the sylvatic interface where dogs were used for hunting purposes. To collect ticks associated with livestock, government and private farms were targeted. To remove ticks from the animals, a Tick Tornado, a forceps-like device designed specifically to remove ticks without harming the host or the tick itself, was used following the manufacturer’s protocol.

Ticks from individual animals were placed into sterile tubes (all ticks removed from one animal in a single tube) and stored at 4 °C during transport. On arrival at The University of West Indies, they were flash frozen at −80 °C. Samples were stored at −80 °C until they were shipped on dry ice to Columbia University for further processing.

### Nucleic acid extraction and species determination

Prior to nucleic acid extraction, ticks (separated according to individual animal source) were each washed in 1 ml of hydrogen peroxide followed by three washes with 1 ml of ultraviolet-irradiated, nuclease-free water and then air-dried. Individual ticks were then transferred into a 1.7 ml microcentrifuge tube containing 100 µl of viral transport media (VTM) (Becton Dickinson) and homogenized. Total nucleic acid (TNA) was extracted from 33 µl of tick homogenate on the EasyMag platform (BioMerieux)^50^ and eluted in 40 µl. From each sample, 11 µl of the TNA was aliquoted for RT-PCR while the remainder was stored at −80 °C.

To identify the tick species, a barcoding PCR was performed using primers targeting the 16s rRNA mitochondrial gene^51^. All PCR products were confirmed using Sanger sequencing.

### Library preparation and genome assembly

Following species confirmation, 33 µl of original VTM homogenate from individual ticks were pooled according to species (n = 20 per pool) to create libraries for high-throughput sequencing (HTS). Before extraction on the EasyMag platform (BioMerieux), 300 µl of pooled material was purified to enrich for viral particles. Pools were filtered (0.45 µM) then treated with RNase A (15 minutes at room temperature) and Turbo DNase and Benzonase (30 minutes at room temperature). This method degrades nucleic acids that are not protected by the presence of a viral capsid. TNA (11 µl) from each tick pool was subjected to first and second-strand cDNA synthesis with Super Script III reverse transcriptase (Invitrogen) and exo- Klenow fragment, respectively.

Double-stranded DNA was mechanically sheared to an average length of 200 nt and purified using the Focused-Ultrasonicator E210 (Covaris, Woburn, MA). Sequencing was performed on the Illumina HiSeq. 4000 system (Illumina, San Diego, CA) using the Hyper Prep kit (KAPA Biosystems, Boston, MA). The demultiplexed FastQ files were adapter trimmed using the cutadapt program (v1.8.3)^52^. Adapter trimming was followed by generation of quality reports using FastQC software (v0.11.5), which were used to determine filtering criteria based on the average quality scores of the reads, presence of indeterminate nucleotides, and homopolymeric reads^53^. The reads were quality filtered and end trimmed with PRINSEQ software (v0.20.3)^54^. Host background levels were determined by mapping filtered reads against a tick reference database (consisting of all *Ixodes scapularis, Ambylomma americanum*, and *Dermacentor variabilis* sequences present in genbank as of June 2018) using Bowtie2 mapper (v2.2.9)^55^. The host-subtracted reads were *de novo* assembled using the MIRA (4.0) and MEGAHIT (1.1.x) assemblers^56,57^. Contigs and unique singletons were subjected to homology search using Megablast against the GenBank nucleotide database. Sequences that showed low or no homology at the nucleotide level were subjected to a BLASTX homology search against the viral GenBank protein database. Sequences from viral BLASTX analysis were submitted to a second round of BLASTX homology search against the complete GenBank protein database to correct for biased E values and taxonomic misassignments. For some viruses present at a low abundance, we only obtained interspersed reads and no contigs. In these cases, we used PCR on cDNA from the virus-positive pool to fill in gaps in the sequence.

### Phylogenetic analysis

Protein sequences were aligned using ClustalW in Geneious 10.2.4. Phylogenetic trees were constructed with MEGA 7.0.26^58^, and the robustness of each node was determined using 1,000 bootstrap replicates using a maximum likelihood (ML) method employing an LG + G + I model with nearest-neighbor interchange (NNI) determined to be the best model through a ML fit of 56 different amino acid substitution models^59^.

### PCR screening

Virus-specific primers were designed for each virus identified through HTS using Primer3Plus. All PCRs were performed using AmpliTaq Gold 360 master mix following the manufacturer’s protocol with the following conditions: heat activation at 95 °C for 10 minutes, 40 cycles of 95 °C for 30 seconds, 55 °C for 30 seconds, 72 °C for 30 seconds, and 72 °C for 5 minutes before storing the samples at 4 °C. The PCR products were visualized on a 1.5% agarose gel with Gel Green. All individual ticks were screened for the presence of each virus identified through HTS. A representative set of PCR products were confirmed using Sanger sequencing.

### Statistical methods

Differences in viral richness among the three tick species were compared by the Kruskal-Wallis test with *post hoc* tests using Bonferroni correction controlling the family-wise error rate at α = 0.05 level. All p-values were two-tailed.

### Ethical approval

All experimental protocols were approved through The University of the West Indies (St. Augustine Campus) ethics committee and in accordance with relevant guidelines and regulations.

## Supplementary information

Supplemental Table 1, Supplemental Table 2

## Data Availability

The complete genome sequences generated during the current study are available in the GenBank repository under accession numbers MN025503:MN025521. The HTS data generated during the current study are available in the NCBI SRA repository under the accession numbers SRR9212027:SRR9212058, https://www.ncbi.nlm.nih.gov/sra/PRJNA546804.

## References

[1] Estrada-Pena A, de la Fuente J (2014). The ecology of ticks and epidemiology of tick-borne viral diseases. Antiviral Res.

[2] Jongejan F, Uilenberg G (2004). The global importance of ticks. Parasitology.

[3] McLean DM (1962). Powassan Virus: Field Investigations in Northern Ontario, 1959 to 1961. Can Med Assoc J.

[4] Puchhammer-Stockl E (1995). Identification of tick-borne encephalitis virus ribonucleic acid in tick suspensions and in clinical specimens by a reverse transcription-nested polymerase chain reaction assay. Clin Diagn Virol.

[5] Hoogstraal H (1979). The epidemiology of tick-borne Crimean-Congo hemorrhagic fever in Asia, Europe, and Africa. J Med Entomol.

[6] Zaki AM (1997). Isolation of a flavivirus related to the tick-borne encephalitis complex from human cases in Saudi Arabia. Trans R Soc Trop Med Hyg.

[7] Topping, N. H., Cullyford, J. S. & Davis, G. E. *Colorado tick fever*. U S Public health service Public Health Reports, Washington,: U.S. Govt. print. off. 1 p. 1., 14 p (1941).

[8] Murthy DP (1958). New virus disease; Kyasanur Forest disease. J Indian Med Assoc.

[9] The Pathology of Louping-Ill and Braxy. *Br Med J*, **1**(2373), 1472–4 (1906). PMC2381604 20762747

[10] Karabatsos N (1978). Supplement to International Catalogue of Arboviruses including certain other viruses of vertebrates. Am J Trop Med Hyg.

[11] Parker J, Plowright W, Pierce MA (1969). The epizootiology of African swine fever in Africa. Vet Rec.

[12] Daubney, R. Nairobi Sheep Disease. *Parasitology*, 10/1931, **23**(4), 507–524.

[13] Zhang YZ (2011). Hemorrhagic fever caused by a novel tick-borne Bunyavirus in Huaiyangshan, China. Zhonghua Liu Xing Bing Xue Za Zhi.

[14] Robles NJC (2018). Epidemiology of severe fever and thrombocytopenia syndrome virus infection and the need for therapeutics for the prevention. Clin Exp Vaccine Res.

[15] Kosoy OI (2015). Novel thogotovirus associated with febrile illness and death, United States, 2014. Emerg Infect Dis.

[16] McMullan LK (2012). A new phlebovirus associated with severe febrile illness in Missouri. N Engl J Med.

[17] Shen S (2018). A novel tick-borne phlebovirus, closely related to severe fever with thrombocytopenia syndrome virus and Heartland virus, is a potential pathogen. Emerg Microbes Infect.

[18] Harvey, E. *et al*. Extensive Diversity of RNA Viruses in Australian Ticks. *J Virol*, **93**(3) (2019). 10.1128/JVI.01358-18 PMC6340049 30404810

[19] Li, C. X. *et al*. Unprecedented genomic diversity of RNA viruses in arthropods reveals the ancestry of negative-sense RNA viruses. *Elife* **4** (2015). 10.7554/eLife.05378 PMC4384744 25633976

[20] Meng, F. *et al*. Virome analysis of tick-borne viruses in Heilongjiang Province, China. *Ticks Tick Borne Dis*, (2018). 10.1016/j.ttbdis.2018.12.002 30583876

[21] Pettersson JH (2017). Characterizing the virome of Ixodes ricinus ticks from northern Europe. Sci Rep.

[22] Souza WM (2018). Viral diversity of Rhipicephalus microplus parasitizing cattle in southern Brazil. Sci Rep.

[23] Tokarz R (2014). Genome characterization of Long Island tick rhabdovirus, a new virus identified in Amblyomma americanum ticks. Virol J.

[24] Tokarz, R. *et al*. Identification of Novel Viruses in Amblyomma americanum, Dermacentor variabilis, and Ixodes scapularis Ticks. *mSphere*, **3**(2) (2018). 10.1128/mSphere.00614-17 PMC5853492 29564401

[25] Tokarz R (2014). Virome analysis of Amblyomma americanum, Dermacentor variabilis, and Ixodes scapularis ticks reveals novel highly divergent vertebrate and invertebrate viruses. J Virol.

[26] Basu, A. K. *Ticks of Trinidad and Tobago an overview*. (London: Academic Press/Elsevier. xv, 89 pages, 2017).

[27] Dantas-Torres F (2010). Biology and ecology of the brown dog tick, Rhipicephalus sanguineus. Parasit Vectors.

[28] Senbill H (2018). Life cycle of the southern cattle tick, Rhipicephalus (Boophilus) microplus Canestrini 1888 (Acari: Ixodidae) under laboratory conditions. Systematic and Applied Acarology.

[29] Camus E, Barre N (1995). Vector situation of tick-borne diseases in the Caribbean Islands. Vet Parasitol.

[30] Gondard M (2017). Ticks and Tick-Borne Pathogens of the Caribbean: Current Understanding and Future Directions for More Comprehensive Surveillance. Front Cell Infect Microbiol.

[31] Sant, C. *et al*. Phylogenetic analysis of Theileria equi and Babesia caballi sequences from thoroughbred mares and foals in Trinidad. *Parasitol Res*, (2019). 10.1007/s00436-019-06240-x 30761425

[32] Georges K (2008). The application of PCR and reverse line blot hybridization to detect arthropod-borne hemopathogens of dogs and cats in Trinidad. Ann N Y Acad Sci.

[33] Sharma A (2010). Erythema Migrans-like Illness among Caribbean Islanders. Emerging Infectious Diseases.

[34] Sayler KA (2014). Isolation of Tacaribe virus, a Caribbean arenavirus, from host-seeking Amblyomma americanum ticks in Florida. PLoS One.

[35] Downs WG (1963). Tacaribe virus, a new agent isolated from Artibeus bats and mosquitoes in Trinidad, West Indies. Am J Trop Med Hyg.

[36] Abudurexiti, A. *et al*. Taxonomy of the order Bunyavirales: update 2019. *Arch Virol* (2019). 10.1007/s00705-019-04253-6 PMC6641860 31065850

[37] Spiegel, M., Plegge, T. & Pohlmann, S. The Role of Phlebovirus Glycoproteins in Viral Entry, Assembly and Release. *Viruses*, **8**(7) (2016). 10.3390/v8070202 PMC4974537 27455305

[38] Shi M (2016). Divergent Viruses Discovered in Arthropods and Vertebrates Revise the Evolutionary History of the Flaviviridae and Related Viruses. J Virol.

[39] Dincer E (2017). Generic amplification and next generation sequencing reveal Crimean-Congo hemorrhagic fever virus AP92-like strain and distinct tick phleboviruses in Anatolia, Turkey. Parasit Vectors.

[40] Qin XC (2014). A tick-borne segmented RNA virus contains genome segments derived from unsegmented viral ancestors. Proceedings of the National Academy of Sciences of the United States of America.

[41] Pascoal, J. O. *et al*. Detection and molecular characterization of Mogiana tick virus (MGTV) in Rhipicephalus microplus collected from cattle in a savannah area, Uberlandia, Brazil. *Ticks Tick Borne Dis*, (2018). 10.1016/j.ttbdis.2018.10.002 30348511

[42] International Committee on Taxonomy of Viruses. and A.M.Q. King, *Virus taxonomy: classification and nomenclature of viruses: ninth report of the International Committee on Taxonomy of Viruses*. (London; Waltham, MA: Academic Press. x, 1327 p, 2012).

[43] Rupprecht C, Kuzmin I, Meslin F (2017). Lyssaviruses and rabies: current conundrums, concerns, contradictions and controversies. F1000Res.

[44] L’vov, D. K. *et al*. *Zoonotic viruses of Northern Eurasia: taxonomy and ecology*. (Amsterdam, Elsevier/Academic Press, xi, 440 pages, 2015).

[45] The Merck veterinary manual. Merck & Co., Inc.: Whitehouse Station, NJ.

[46] Costero A, Grayson MA (1996). Experimental transmission of Powassan virus (Flaviviridae) by Ixodes scapularis ticks (Acari:Ixodidae). Am J Trop Med Hyg.

[47] Mansfield KL (2017). Emerging Tick-Borne Viruses in the Twenty-First Century. Front Cell Infect Microbiol.

[48] Emmerich P (2018). Viral metagenomics, genetic and evolutionary characteristics of Crimean-Congo hemorrhagic fever orthonairovirus in humans, Kosovo. Infect Genet Evol.

[49] Ladner JT (2016). A Multicomponent Animal Virus Isolated from Mosquitoes. Cell Host Microbe.

[50] Loens K (2007). Evaluation of NucliSens easyMAG for automated nucleic acid extraction from various clinical specimens. Journal of Clinical Microbiology.

[51] Black WCt, Piesman J (1994). Phylogeny of hard- and soft-tick taxa (Acari: Ixodida) based on mitochondrial 16S rDNA sequences. Proc Natl Acad Sci USA.

[52] Macel M (2011). Cutadapt removes adapter sequences from high-throughput sequencing reads. EMBnet.journal.

[53] S, A., FastQC: a quality control tool for high throughput sequence data (2010).

[54] Schmieder R, Edwards R (2011). Quality control and preprocessing of metagenomic datasets. Bioinformatics.

[55] Langmead B, Salzberg SL (2012). Fast gapped-read alignment with Bowtie 2. Nat Methods.

[56] Chevreux B, Wetter T, Suhai S (1999). Genome sequence assembly using trace signals and additional sequence information. Comput Sci Biol.

[57] Li D (2015). MEGAHIT: an ultra-fast single-node solution for large and complex metagenomics assembly via succinct de Bruijn graph. Bioinformatics.

[58] Kumar S, Stecher G, Tamura K (2016). MEGA7: Molecular Evolutionary Genetics Analysis Version 7.0 for Bigger Datasets. Mol Biol Evol.

[59] Le SQ, Gascuel O (2008). An improved general amino acid replacement matrix. Mol Biol Evol.

