# Supplementary material for: Viral Diversity of Tick Species Parasitizing Cattle and Dogs in Trinidad and Tobago
Source: Sci Rep. 2019 Jul 18;9:10421. doi: 10.1038/s41598-019-46914-1 (PMC6639388; doi:10.1038/s41598-019-46914-1)
Supplement: Supplementary file 1 — Supplemental Table 1, Supplemental Table 2 [file 41598_2019_46914_MOESM1_ESM.pdf]

Viral Diversity of Tick Species Parasitizing Cattle and Dogs in Trinidad and Tobago

Stephen Sameroff *[1, 2], Rafal Tokarz[1], Roxanne Charles[2], Komal Jain[1], Alexandra Oleynik[1], Xiaoyu Che[1], Karla Georges[2], Christine Carrington[3], W. Ian Lipkin [1], and Chris Oura [2]

[1] Center for Infection and Immunity, Mailman School of Public Health, Columbia University, New York, New York, USA
[2] School of Veterinary Medicine, The University of the West Indies, St. Augustine, Trinidad and Tobago
[3] Department of Preclinical Sciences, The University of the West Indies, St. Augustine, Trinidad and Tobago

Supplemental Table 1: Read count summary for the individual pools

Sample ID	# of Raw Reads	# of Primer Trimmed Reads	# of Reads after Filtration	% Removed after Filtration	# of Reads after Host Subtraction	% Removed after Host Subtraction	# Used in Assembly	% Used in Assembly	# of Contigs generated	Longest Contig Length	# of Singletons	# of Unique Singletons
TTP-Pool-001	15605902	15378897	14636867	4.82%	3660188	74.99%	1036578	28.32%	127118	4344	2623611	1728230
TTP-Pool-002	16994769	16811669	15986149	4.91%	4860887	69.59%	1530751	31.49%	220892	5629	3330139	2287441
TTP-Pool-003	18975693	18763388	18079826	3.64%	6816586	62.30%	2247887	32.98%	306002	2936	4568702	2787126
TTP-Pool-004	17674288	17495764	16912486	3.33%	4575800	72.94%	1431843	31.29%	211587	3510	3143960	2118612
TTP-Pool-005	20745591	20520569	19701348	3.99%	5584991	71.65%	1656973	29.67%	246954	2650	3928021	2725185
TTP-Pool-006	18514550	18198835	17062512	6.24%	5152599	69.80%	1650199	32.03%	239037	4210	3502402	2527450
TTP-Pool-007	14085988	13891544	13341130	3.96%	3788081	71.61%	1217667	32.14%	178273	4351	2570417	1748460
TTP-Pool-008	24978926	24507793	23370103	4.64%	7180293	69.28%	2137973	29.78%	324922	4021	5042323	3668536
TTP-Pool-009	12817458	12016789	4705593	60.84%	2962463	37.04%	730581	24.66%	94800	3797	2231883	1838212
TTP-Pool-010	21477328	21176274	20125657	4.96%	5993040	70.22%	1742134	29.07%	256774	2657	4250910	2896080
TTP-Pool-011	24729418	24313151	23152129	4.78%	7010957	69.72%	2062998	29.43%	306733	3221	4947962	3416411
TTP-Pool-012	20993882	20662063	19761697	4.36%	5892883	70.18%	1758706	29.84%	260707	4615	4134186	2847819
TTP-Pool-013	17763768	17521076	16525051	5.68%	4950870	70.04%	1522519	30.75%	223303	3328	3428355	2385875
TTP-Pool-014	58589756	57157129	51930496	9.14%	15555665	70.05%	4828968	31.04%	695486	6308	10726712	7460495
TTP-Pool-015	21003881	20672939	19654473	4.93%	5627420	71.37%	1731628	30.77%	252953	5454	3895792	2645842
TTP-Pool-016	20843472	20174228	17939082	11.08%	5812438	67.60%	2245068	38.63%	304666	2772	3567377	2802755
TTP-Pool-017	26519936	26164675	25790727	1.43%	1949041	92.44%	620881	31.86%	63581	7882	1328160	749188
TTP-Pool-018	17076939	16880962	16394435	2.88%	1766141	89.23%	515284	29.18%	48641	5498	1250857	856085
TTP-Pool-019	18255330	18097482	17912185	1.02%	1735302	90.31%	612424	35.29%	47859	4212	1122878	596950
TTP-Pool-020	20792418	20580353	20330326	1.21%	1807371	91.11%	558894	30.92%	53575	6977	1248478	788425
TTP-Pool-021	17214852	17060965	16854688	1.21%	1347423	92.01%	426986	31.69%	44654	3105	920437	545636
TTP-Pool-022	15385358	15249162	15086804	1.06%	1111718	92.63%	410942	36.96%	36555	3584	700777	384166
TTP-Pool-023	17928438	17666815	17266011	2.27%	2494418	85.55%	710596	28.49%	76216	3339	1783823	1360840
TTP-Pool-024	18003462	17820154	17608164	1.19%	1307406	92.58%	436464	33.38%	44535	3870	870942	497544
TTP-Pool-025	13743185	13514004	13283041	1.71%	1619136	87.81%	431528	26.65%	45139	6505	1187608	950397
TTP-Pool-026	16218788	16085450	15873896	1.32%	1314959	91.72%	431252	32.80%	41350	3008	883707	600654
TTP-Pool-027	13123316	13004528	12860895	1.10%	762296	94.07%	272915	35.80%	26123	4982	489381	264634
TTP-Pool-028	11195244	11083213	10959402	1.12%	711987	93.50%	250689	35.21%	24040	3462	461298	254484
TTP-Pool-029	15733460	15411741	15062646	2.27%	2056799	86.35%	544581	26.48%	55519	7245	1512218	1259401
TTP-Pool-030	27759260	27459386	27064547	1.44%	1988865	92.65%	675215	33.95%	61905	5943	1313650	722122
TTP-Pool-031	17571564	17364160	17125644	1.37%	1255968	92.67%	414820	33.03%	41193	7597	841148	509459
TTP-Pool-032	33994101	33458618	32800080	1.97%	2385615	92.73%	804865	33.74%	81179	3718	1580750	887685

Supplemental Table 2: Blast hit summary by Kingdom for the individual pools

Sample Name	Viruses	Phages	Primates	Mammals	Rodents	Vertebrates	Invertebrates	Bacteria	Plants	Environmental Samples	Synthetic	Unassigned	N/A	Total Blast Count	Total Blast %	No Hits Count	No Hits %
TTP-Pool-1	284	1	646	295	454	12699	666755	27559	20618	30568	126	2	19	760026	40.96%	1095316	59.04%
TTP-Pool-2	372	4	549	561	1425	18547	1341776	4474	68964	36925	143	0	63	1473803	58.76%	1034528	41.24%
TTP-Pool-3	8973	7	611	1304	1586	16903	1355943	3214	50133	59009	89	0	43	1497815	48.42%	1595312	51.58%
TTP-Pool-4	334	6	665	654	1459	21327	1322726	3225	63960	41486	76	0	46	1455964	62.48%	874231	37.52%
TTP-Pool-5	381	8	2781	3218	1898	28055	1555135	4136	73585	39265	97	1	47	1708607	57.49%	1263530	42.51%
TTP-Pool-6	250	6	734	855	1305	19363	1359048	4067	63707	40150	97	1	51	1489634	53.85%	1276857	46.15%
TTP-Pool-7	306	6	4040	2305	1401	21874	1044649	1817	47772	25694	77	0	22	1149963	59.69%	776762	40.32%
TTP-Pool-8	369	8	1433	3640	1798	37045	2069219	3134	86865	55401	107	0	67	2259086	56.57%	1734371	43.43%
TTP-Pool-9	63	17	1408	3015	103	6670	84548	284761	2772	108305	3231	61	324	495278	25.62%	1437736	74.38%
TTP-Pool-10	428	4	3238	2894	1947	30447	1589950	4756	74713	48634	122	1	58	1757192	55.73%	1395655	44.27%
TTP-Pool-11	370	9	1228	1242	2195	30580	1920537	4316	87397	60117	93	0	45	2108129	56.62%	1615018	43.38%
TTP-Pool-12	415	6	2134	1248	2085	33894	1657177	3085	69172	51077	96	1	33	1820423	58.56%	1288097	41.44%
TTP-Pool-13	297	7	1004	1348	1392	25773	1300843	3756	63098	35720	97	1	41	1433377	54.94%	1175801	45.06%
TTP-Pool-14	687	10	1698	2414	2870	48920	3805459	37219	165870	144709	356	6	227	4210445	51.62%	3945516	48.38%
TTP-Pool-15	552	2	6583	2236	2332	29421	1537697	3061	66483	51042	107	1	46	1699563	58.63%	1199235	41.37%
TTP-Pool-16	278	10	724	1054	719	16134	1392060	21334	50822	52449	236	1	94	1535915	49.43%	1571498	50.57%
TTP-Pool-17	286	18	779	9819	171	2281	470984	3063	8325	15858	8	2	396	511990	63.82%	290243	36.18%
TTP-Pool-18	7	15	2269	537	143	1355	290723	23072	4641	16723	6	25	311	339827	37.91%	556485	62.09%
TTP-Pool-19	2381	9	261	14959	129	1253	332605	2840	5833	11491	3	8	231	372003	58.30%	266125	41.70%
TTP-Pool-20	328	5	275	22719	113	1336	378337	5065	5819	11583	2	1	237	425820	51.09%	407685	48.91%
TTP-Pool-21	218	9	257	10025	92	1298	339081	1015	5655	11561	6	1	202	369420	63.29%	214289	36.71%
TTP-Pool-22	275	8	307	3777	67	1121	275228	1502	4950	8796	2	1	233	296267	71.25%	119558	28.75%
TTP-Pool-23	203	10	863	25537	152	1875	430617	1550	8089	10602	5	0	183	479686	33.72%	942936	66.28%
TTP-Pool-24	234	6	939	4280	97	1178	329431	1385	5443	10874	6	0	237	354110	66.17%	181029	33.83%
TTP-Pool-25	223	15	803	43448	161	1320	264033	2378	4060	8610	3	1	193	325248	32.93%	662458	67.07%
TTP-Pool-26	227	15	1388	24258	91	1568	276701	2233	4572	9492	5	3	175	320728	50.56%	313629	49.44%
TTP-Pool-27	172	11	113	387	56	748	204514	1352	3554	7751	1	0	210	218869	76.26%	68151	23.74%
TTP-Pool-28	256	11	96	185	48	848	196234	1097	3339	7273	1	0	182	209570	76.08%	65904	23.92%
TTP-Pool-29	383	17	462	687	145	1480	290131	2321	3863	9222	8	0	210	308929	23.67%	996180	76.33%
TTP-Pool-30	566	21	283	967	203	2044	434670	8573	6439	16089	5	1	375	470236	60.84%	302657	39.16%
TTP-Pool-31	428	12	194	400	87	1439	300913	854	4709	10326	2	0	250	319614	58.78%	224149	41.22%
TTP-Pool-32	378	18	633	651	170	2768	561159	1496	9082	22055	10	0	357	598777	62.87%	353618	37.13%
